# Wild‐type FLT3 and FLT3 ITD exhibit similar ligand‐induced internalization characteristics

**DOI:** 10.1111/jcmm.15132

**Published:** 2020-03-10

**Authors:** Fabienne Kellner, Andreas Keil, Katrin Schindler, Todor Tschongov, Kerstin Hünninger, Hannah Loercher, Peter Rhein, Sylvia‐Annette Böhmer, Frank‐D. Böhmer, Jörg P. Müller

**Affiliations:** ^1^ Institute for Molecular Cell Biology Center for Molecular Biomedicine Jena University Hospital Jena Germany; ^2^ Fungal Septomics Leibniz Institute for Natural Product Research and Infection Biology Hans Knöll Institute Jena Germany; ^3^ Luminex B.V. ‘s‐Hertogenbosch The Netherlands

**Keywords:** degradation, Fms‐like tyrosine kinase 3 internal tandem duplications, GFP hybrid genes, oncogene, plasma membrane, receptor endocytosis, receptor tyrosine kinase

## Abstract

Class III receptor tyrosine kinases control the development of hematopoietic stem cells. Constitutive activation of FLT3 by internal tandem duplications (ITD) in the juxtamembrane domain has been causally linked to acute myeloid leukaemia. Oncogenic FLT3 ITD is partially retained in compartments of the biosynthetic route and aberrantly activates STAT5, thereby promoting cellular transformation. The pool of FLT3 ITD molecules in the plasma membrane efficiently activates RAS and AKT, which is likewise essential for cell transformation. Little is known about features and mechanisms of FLT3 ligand (FL)‐dependent internalization of surface‐bound FLT3 or FLT3 ITD. We have addressed this issue by internalization experiments using human RS4‐11 and MV4‐11 cells with endogenous wild‐type FLT3 or FLT3 ITD expression, respectively, and surface biotinylation. Further, FLT3 wild‐type, or FLT3 ITD‐GFP hybrid proteins were stably expressed and characterized in 32D cells, and internalization and stability were assessed by flow cytometry, imaging flow cytometry, and immunoblotting. FL‐stimulated surface‐exposed FLT3 WT or FLT3 ITD protein showed similar endocytosis and degradation characteristics. Kinase inactivation by mutation or FLT3 inhibitor treatment strongly promoted FLT3 ITD surface localization, and attenuated but did not abrogate FL‐induced internalization. Experiments with the dynamin inhibitor dynasore suggest that active FLT3 as well as FLT3 ITD is largely endocytosed via clathrin‐dependent endocytosis. Internalization of kinase‐inactivated molecules occurred through a different yet unidentified mechanism. Our data demonstrate that FLT3 WT and constitutively active FLT3 ITD receptor follow, despite very different biogenesis kinetics, similar internalization and degradation routes.

## 1 INTRODUCTION

Fms‐like tyrosine kinase 3 (FLT3) is a class III receptor tyrosine kinase (RTK) which plays a role in proliferation and differentiation of B‐cell progenitors, myelomonocytic and dendritic cells, as well as in the maintenance of pluripotent haematopoietic stem cells (reviewed in Toffalini and Demoulin, 2010).^1^ Activating mutations of FLT3, either in the form of internal tandem duplication (ITD) mutations in the juxtamembrane (JM) domain or point mutations in the tyrosine kinase domain, are frequently reported in acute myeloid leukaemia (AML). Both types of mutations are believed to causally contribute to leukaemogenesis.^2^ Internal tandem duplications (ITD) of FLT3 occur in approximately one fourth of AML cases and induce ligand‐independent constitutive signalling. FLT3 ITD is associated with high relapse rates and poor overall survival of AML patients.

The resting FLT3 protein at the cell surface is activated via its cognate ligand FL (FLT3 ligand). Important steps of activation include the phosphorylation of the tyrosine‐sites Y589, Y591 and Y599 of the JM segment, abolishing its cis‐autoinhibitory function and presumably resulting in binding of Src‐family kinases. FL‐mediated phosphorylation of Y768, Y955 and Y969 mediates Grb2 binding and the association of the scaffolding protein Gab2, which in turn directly interacts with PI3K mediating activation of the AKT signalling pathway.^3^ In parallel, stimulation of FLT3 mediates activation of mitogen‐activated protein kinases ERK1/2.^4^, ^5^

Ligand‐induced activation of RTK also triggers negatively regulatory mechanisms that lead to the termination of signalling. Upon ligand‐mediated receptor activation, c‐Cbl, a ubiquitin E3 ligase, is recruited and mediates RTK ubiquitination and subsequent internalization. Inhibition of c‐Cbl function by mutations causing loss of E3 activity severely disturbed the negative regulation of FLT3 signal transduction by blocking FLT3 internalization and ubiquitination resulting in transforming signalling of FLT3.^6^

The current knowledge on the biogenesis of FLT3 is mainly based on general insights in RTK maturation.^4^ The wild‐type (WT) FLT3 protein is co‐translationally translocated into the endoplasmatic reticulum (ER). Here the luminal‐faced N‐terminus of the receptor undergoes multistep glycosylation and folding, as mediated by the ER luminal enzyme machinery.^7^ The ER quality control system ensures that only folded and complex glycosylated FLT3 molecules are transported via the Golgi system to the plasma membrane.^8^ While the FLT3 WT can be predominantly found as a mature, complex glycosylated 150 kDa molecule, FLT3 ITD exists mainly in an immature, high‐mannose 130 kDa form.^8^ Abnormal signalling of FLT3 ITD appears tightly linked to its aberrant localization. The intracellular pool of FLT3 ITD effectively activates STAT5 and upregulates its targets, Pim‐1/2, but ineffectively activates PI3K/AKT and RAS/MAPK pathways. In contrast, the pool of FLT3 ITD molecules in the plasma membrane efficiently activates RAS and AKT.^9^, ^10^, ^11^

Intracellular retention depends on FLT3 ITD kinase activity. An inactivating K644A point mutation of FLT3 ITD, treatment with FLT3 kinase inhibitors or overexpression of protein‐tyrosine phosphatases promoted surface localization. Thus, prerequisite of the intracellular retention is the constitutive activity of the receptor mediating slowdown of its post‐translational biogenesis.^8^, ^12^ In an “intracellular active kinase load” model Chan suggested that recruitment of phosphotyrosine‐binding domain‐containing proteins causes the retardation,^13^ but the molecular mechanism of FLT3 ITD retention in intracellular compartments is currently still not known.

Dormant RTKs reside in the cell membrane as autoinhibited monomers. Dimerization or oligomerization of receptor tyrosine kinases is an immediate consequence of their cognate ligand binding causing trans‐phosphorylation within the dimer and RTK activation.^14^ Structural characteristics of wild‐type FLT3‐dimer formation as consequence of FL binding have recently been elucidated.^15^, ^16^ Interestingly, regardless of the sequence variety of JM domain, FLT3 ITD receptors form homodimers in absence of FL and, if co‐transfected with FLT3 WT, FLT3 WT/FLT3 ITD heterodimers.^17^, ^18^ Thus, elongation mutations of the JM domain promote FLT3 ITD receptor dimerization and its subsequent phosphorylation and auto‐activation in the absence of the ligand.^17^

Endocytosis, stability or recycling of surface FLT3 ITD, and the contribution of possible alterations compared with WT FLT3 to the aberrant localization have previously not been investigated.^8^

To get further insight into these issues, we have comparatively analysed FLT3 WT and FLT3 ITD internalization, stability and effect of kinase activity in human cell lines and 32D cell lines expressing FLT3‐GFP hybrid proteins. FL‐stimulated internalization and protein stability of WT FLT3 and FLT3 ITD were very similar and followed, at least in part, a clathrin‐dependent mode. Internalization was attenuated by kinase inactivation and kinase‐independent internalization occurred not through a clathrin‐dependent mechanism. These observations indicate that FLT3 ITD is using similar endocytosis and degradation routes as the FLT3 WT receptor.

## 2 MATERIAL AND METHODS

### 2.1 Antibodies

The antibodies used in the study are listed in the Supporting information.

### 2.2 Cell lines

RS4‐11 cells and MV4‐11 cells were obtained from German Collection of Microorganisms and Cell Cultures GmbH (Braunschweig, Germany) and were maintained as previously described.^11^ The FLT3‐GFP hybrid genes were lentivirally packed and subsequently transduced in 32D cells. Cells were sorted for similar GFP production. Further details are outlined in the Supporting information.

### 2.3 Signalling analyses

Immunoprecipitation, immunoblotting and signalling analyses were carried out with standard methods as described previously.^8^ Further details are given in the Supporting information.

### 2.4 Cell proliferation

For analysis of proliferation, 32D cells were harvested, washed with PBS and cultured for 5 days in RPMI medium without cytokines, with FL (100 ng/mL), or IL‐3 as indicated. For cell quantification 90 μL of cells were supplemented with 10 μL counting beads (1000 beads/μL). The ratio between the cells number and bead number was examined by flow cytometry.

### 2.5 Biotinylation assay

32D cells were harvested and surface proteins were labelled with biotin by incubating cells in 400 μL Sulfo‐NHS‐biotin (1 mg/mL PBS) on a rotator for 1 hour at 4°C. Unbound biotin was subsequently removed by two washing steps with 1 mL ice‐cold PBS. Cells were lysed and protein concentration was determined as described previously. Biotinylated proteins were precipitated with NeutrAvidin beads overnight at 4°C. On the next day beads were washed three times and supernatant was completely removed and processed for immunoblotting.

To quantify the amount of internalized receptor a biotinylation surface removal according to Joffre^19^ was used. Briefly, cell surface proteins were labelled Sulpho‐NHS‐biotin for 1 hour at 4°C prior receptor stimulation as described above. After stimulation with 100 ng/mL FL, cells were washed two times with 1 mL ice‐cold PBS (containing 0.5% BSA). Remaining surface‐localized biotin was removed by incubating cells with the reducing agent MesNa (100 mmol/L sodium 2‐mercaptoethanesulfonate). Residual MesNa was inactivated by incubation with 25 mmol/L sodium iodoacetate (IAA) in PBS with 0.5% BSA for 10 minutes on ice. Subsequently cells were washed twice with 1 mL ice‐cold PBS with 0.5% BSA, lysed and further processed for NeutrAvidin precipitation of internalized biotinylated FLT3.

### 2.6 Immunocytochemical analysis of FLT3 in 32D cells

Immunofluorescence labelling was performed according to standard procedures as previously described.^10^ In brief, cells were seeded on poly‐lysine coated slides, starved in cytokine and serum‐free medium for 4 hours and treated with cycloheximide (20 μg/mL) for 90 minutes. The plasma membrane of 32D cells was stained on ice with WGA conjugated to Alexa Fluor 633 (Molecular Probes) for 5 minutes. GFP fluorescence was used as readout for localization of FLT3 proteins. Images were obtained with the Zeiss laser scanning microscope 510 and processed with imaging software ZEN (Carl Zeiss Jena).

### 2.7 Analysis of FLT3 degradation

Cells were washed and further incubated in RPMI medium with FCS (10%) without cytokines. Cells were incubated with cycloheximide (20 μg/mL) for 30 minutes at 37°C prior incubation with FL (100 ng/mL) or left untreated. At indicated time points after addition of FL, the amount of FLT3‐GFP per cell was analysed by flow cytometry via GFP fluorescence. Alternatively, levels of FLT3 protein were analysed by immunoblotting of cell lysates.

### 2.8 Flow cytometry

Flow cytometry was carried out under standard conditions as described in the Supporting information. Cell membrane‐localized FLT3/FLT3 ITD was stained from vital cells. Fixed and permeabilized cells were used for staining of total FLT3/FLT3 ITD.

### 2.9 Imaging flow cytometry

A 12 channel Amnis^®^ brand ImageStreamX^®^ Mark II (Luminex) imaging flow cytometer was used for visualization of subcellular localization of FLT3‐GFP proteins. For detection of co‐localization with the cell membrane receptor cell were stained with WGA‐Alexa Fluor 633. Details about data processing are described in the Supporting information.

## 3 RESULTS

### 3.1 Surface biotinylation reveals similar features of FLT3 and FLT3 ITD internalization in human cell lines

**Figure 1:**
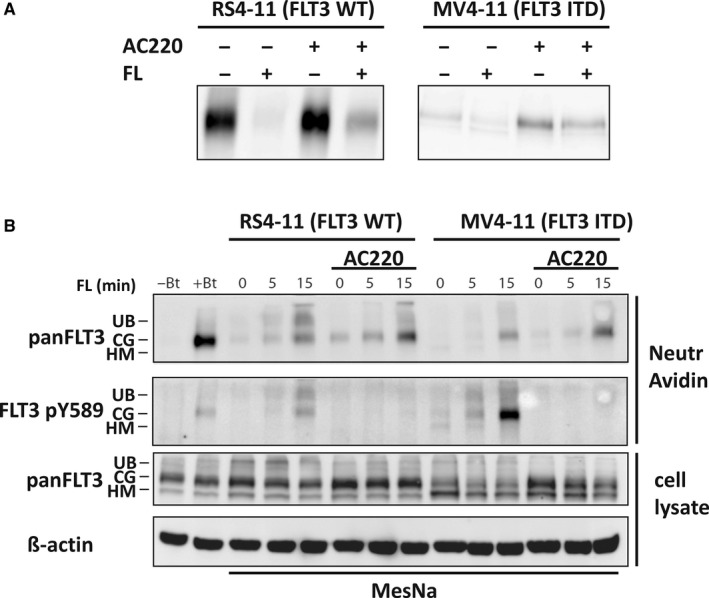
Ligand‐mediated internalization of FLT3 proteins in human cells. A, FLT3 WT expressing RS4‐11 and FLT3 ITD expressing MV4‐11 cells were incubated for 2 h with 10 nmol/L AC220 or left untreated. Cells were stimulated with 100 ng/mL FL for 15 min. Cells were treated with NHS‐biotin to label FLT3 located at the cell surface. After cell lysis, biotinylated FLT3 was precipitated with NeutrAvidin beads. The amount of biotinylated FLT3 was analysed by immunoblotting. A representative blot of several repeated experiments is presented. B, Kinase‐independent internalization of biotin‐labelled FLT3‐WT and FLT3‐ITD. RS4‐11 and MV4‐11 cells were treated with 10 nmol/L AC220 for 2 h or remained untreated. Afterwards, cells were incubated for 1 h at 4°C with membrane‐impermeable NHS‐biotin and stimulated for 5 and 15 min with 100 ng/mL FL or were left unstimulated. Subsequently cells were treated with MesNa and thereafter with IAA to remove the biotin label from the surface pool of FLT3. IAA was required to inactivate the excess of MesNa. Thereafter, cells were lysed and the intracellular, biotinylated FLT was precipitated with NeutrAvidin beads and analysed by immunoblotting. Total lysate aliquots were analysed for comparison. − Bt, negative precipitation fraction contained cells without NHS‐biotin, MesNa and IAA treatment; + Bt, positive precipitation fraction included cells that were treated with NHS‐biotin and without MesNa and IAA

RS4‐11 and MV4‐11 cells are human leukaemia cell lines expressing endogenously similar levels of FLT3 WT or FLT3 ITD, respectively. As shown previously^11^ FLT3 proteins in these cell lines are differently localized, with WT FLT3 mainly at the cell surface, and FLT3 ITD largely localized intracellularly. To investigate ligand‐induced internalization of surface‐bound FLT3 or FLT3 ITD, we followed previously published protocols of cell surface protein biotinlyation, which were used earlier for studies on RTK internalization and recycling including of the class III RTK PDGFR.^19^, ^20^, ^21^ To assess the role of FLT3 kinase activity, cells were pre‐incubated with the selective FLT3 kinase inhibitor AC220 (quizartinib), which abrogated FLT3 phosphorylation completely (Figure S1). Surface proteins were labelled covalently using membrane‐impermeable biotinylation, cells were ligand stimulated and biotinylated proteins were subsequently isolated by Neutravidin precipitation and FLT3 was detected by immunoblotting. As shown in Figure 1A, resident WT FLT3 was abundantly detectable at the surface of RS4‐11 cells. Ligand stimulation strongly reduced the amount of labelled FLT3, consistent with effective internalization. Similar observations were made in AC220‐treated cells, although the extent of internalization appeared somewhat attenuated. In MV4‐11 cells, only very weak signals of surface FLT3 ITD were captured, however clearly more surface‐bound FLT3 ITD was detected after AC220 treatment. Ligand stimulation of AC220‐treated cells led to decreased signals consistent with FLT3 ITD internalization. To detect the internalized FLT3 directly, a modification of the method was used by treating the cells subsequent to FL stimulation with the membrane‐impermeable compound sodium 2‐mercaptoethanesulfonate (MesNa), which is removing the remaining surface biotinylation for example of not internalized FLT3 species. Subsequently, cell lysates were generated, biotinylated proteins isolated by Neutravidin precipitation as described and FLT3 was detected by immunoblotting. As shown in Figure 1B, resident WT FLT3 in RS4‐11 cells is effectively internalized upon ligand stimulation in a time‐dependent manner. AC220, while abrogating all FLT3 phosphorylation, still allowed effective internalization. FLT3 ITD in MV4‐11 cells was also internalized in a ligand‐dependent manner. Signals of internalized receptor were comparatively weak, which likely relates to low surface levels in untreated cells. AC220 treatment appeared to augment internalization of FLT3 ITD, presumably because inhibitor treatment caused higher amounts of FLT3 ITD at the cell surface to be accessible for biotinylation. Still, time‐dependent FL‐induced internalization can be robustly detected.

Taken together, these assays revealed similar features for internalization of WT FLT3 and FLT3 ITD upon ligand stimulation and showed robustly also a certain amount of kinase‐independent internalization. A limitation of these assays is their qualitative character. We therefore went on with alternative assays to assess the internalization process in a more quantitative manner.

### 3.2 Novel 32D cell lines expressing FLT3‐GFP fusion proteins exhibit subcellular localization of FLT3 proteins consistent with earlier findings

**Figure 2:**
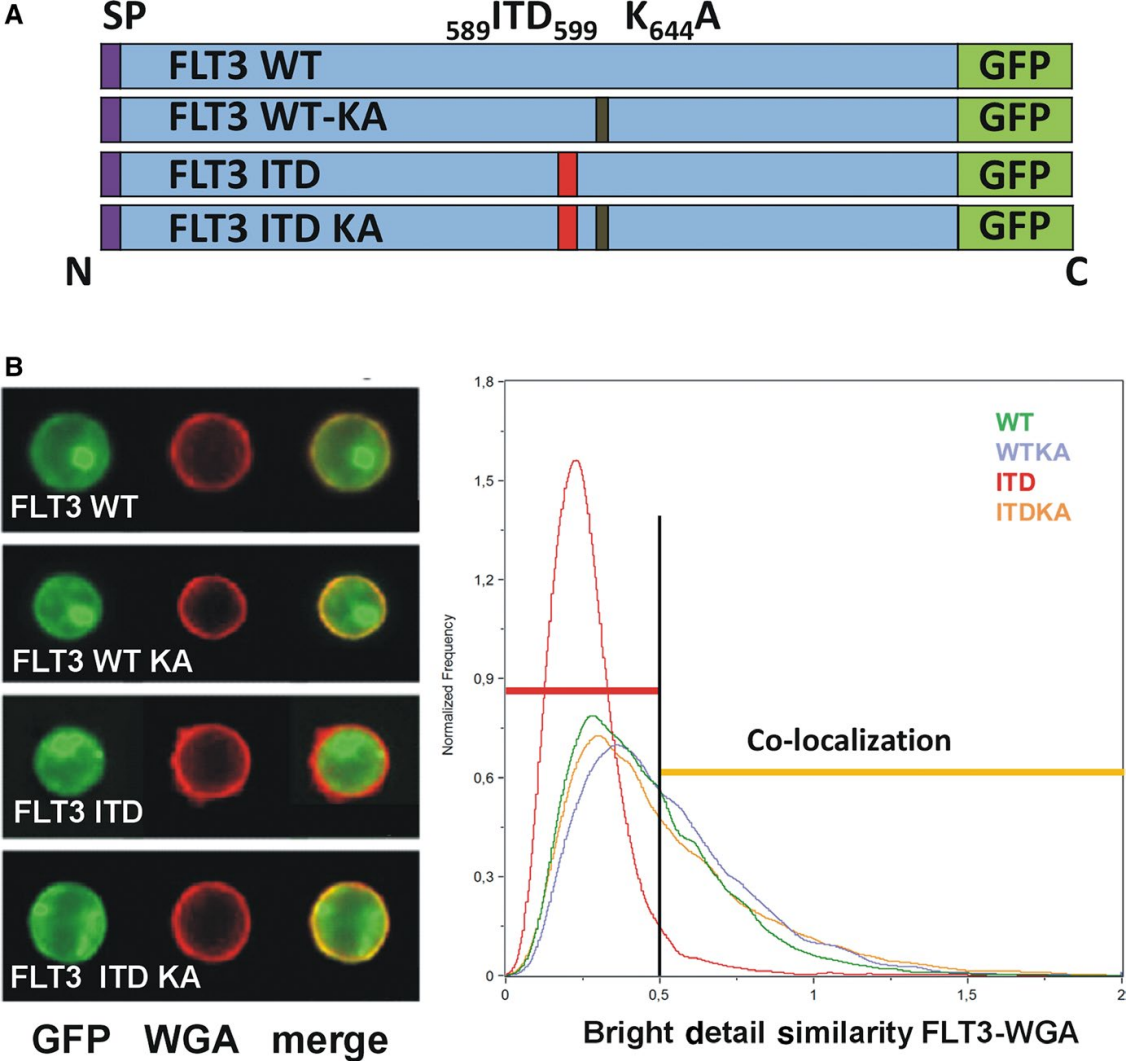
FLT3‐GFP hybrid proteins. A, Scheme of FLT3‐GFP hybrid proteins used in the study. ITD, internal tandem duplication in the JM domain; K_644_A, mutation in the kinase domain resulting in inactivation of the kinase activity; SP, signal peptide. B, Imaging flow cytometry of FLT3‐GFP proteins. 32D cells expressing indicated FLT3‐variants were treated for 1.5 h with 20 μg/mL cycloheximide (CHX). Subsequently, cells were stained 5 min with WGA‐Alexa Fluor 663 to mark the plasma membrane. Example images of cells demonstrating localization of FLT3‐GFP, WGA‐Alexa Fluor 633 and merged images. Graphs were obtained by analysis of fluorescence images of 10^4^ cells with IDEAS software. After gating for GFP WGA‐Alexa Fluor 633‐positive living single cells, co‐localization of GFP with WGA‐stained cell membrane was calculated (Bright Detail Similarity FLT3‐WGA). Threshold of 0.5 marks limit of co‐localization (<0.5 no co‐localization, >0.5 co‐localization)

To allow a better quantitative characterization of internalization processes, we generated novel 32D cell lines expressing FLT3 WT and FLT3 ITD‐GFP fusion proteins. Genes encoding human FLT3 WT or FLT3 ITD in lentiviral vectors were 3’‐terminally fused in frame to eGFP. To study the role of the receptor activity/ phosphorylation, kinase‐inactive FLT3 WT K_644_A‐ and FLT3 ITD K_644_A‐GFP hybrid genes were also generated (see schema Figure 2A). The constructs were packed into lentiviral particles and transduced into 32D cells. Populations of cells expressing similar levels of GFP were subsequently sorted using flow cytometry. Thus, cellular GFP fluorescence of 32D cell reflected the level of FLT3. Detailed characterization of these cell lines indicated that signalling qualities of the GFP‐tagged FLT3 proteins were similar to their untagged counterparts,^8^, ^9^, ^10^, ^22^, ^23^ and biological responses were likewise as expected (Figure S2). Expression of FLT3 ITD in 32D cells resulted in cytokine‐independent growth, whereas kinase‐inactive FLT3 KA proteins remained cytokine‐dependent comparable to un‐transduced 32D cells (Figure S2A). Incubation of cell lines with IL‐3 resulted in efficient proliferation of the cell populations. Similarly, clonal growth of 32D cells in methylcellulose revealed cytokine‐independent cell transformation of FLT3‐ITD expressing cells only (data not shown).

Incubation of 32D FLT3 WT cells with FL enhanced FLT3 phosphorylation and appearance of a high molecular weight, presumably ubiquitinated form as shown previously,^22^ but a basic cytokine‐independent phosphorylation of mature FLT3 WT could be observed, which may have possibly been caused by the C‐terminal GFP fusion, but did not result in appreciable downstream signalling. FLT3 ITD showed efficient constitutive phosphorylation of complex glycosylated mature as well as immature high mannose form in the absence of its cytokine (Figure S2B). No phosphorylation of FLT3 WT KA or FLT3 ITD KA proteins could be observed. FL stimulation of 32D FLT3 WT cells resulted in activation of phosphorylation of ERK1/2 and AKT (Figure S2B). FLT3 ITD expressing 32D cells showed the expected activation of STAT5 even in the absence of cytokines. No activation of FLT3 downstream signal transduction could be observed in 32D cell expressing FLT3 WT KA or FLT3 ITD KA in response to FL treatment. Taken together, characterization of 32D cells stably expressing FLT3 GFP hybrid proteins demonstrated the expected functionality of FLT3 WT, oncogenic FLT3 ITD and inactive FLT3 KA receptor variants. FLT3.

We further characterized the localization of the FLT3‐GFP hybrid proteins using laser scanning microscopy (Figure S2C). While GFP signals were used for localization of receptor molecules, staining with WGA‐Alexa Fluor 633‐conjugate labelled the plasma membrane. Co‐localization of GFP and Alexa Fluor 633 was demonstrated for FLT3 WT and FLT3 KA proteins. Staining of active FLT3 ITD was intercellular, whereas kinase‐inactive FLT3 ITD localized to the cell surface (Figure S2C). To obtain quantitative imaging data, we used the power of imaging flow cytometric analysis.^24^ Co‐localization of FLT3‐GFP with WGA‐Alexa Fluor 633 conjugate was analysed for 1‐2 × 10^4^ cells. These data confirmed the predominant surface localization of WT FLT3 receptor and of enzymatically inactive KA forms, but the intracellular retention of constitutively active FLT3 ITD (Figure 1B). To assess localization of the FLT3 proteins further with an alternative quantitative method, non‐permeabilized cells were labelled with CD135‐PE antibody. The ratio of bound CD135‐PE to cellular GFP level further confirmed efficient surface expression of the hybrid proteins with exception of active FLT3 ITD (Figure S1D). The amount of membrane‐localized receptor (PE‐signal) was also calculated in relation to total receptor level detected in fixed and permeabilized cells. Quantities of surface‐localized FLT3 were similar for FLT3 WT, FLT3 WT KA as well as FLT3 ITD KA protein, but were diminished in FLT3 ITD cells (Figure S2E).

Taken together, FLT3‐GFP hybrid proteins in the newly generated 32D cell pools revealed activity and localization features as they were found previously for endogenous receptors and in different model systems^8^, ^10^, ^11^ despite their GFP epitope‐tagging. Importantly, mislocalization of active FLT3 ITD was confirmed for the first time by quantitative analysis using imaging flow cytometry. These cell lines appeared therefore suitable to further study FLT3 internalization.

### 3.3 FL‐mediated internalization of FLT3 is only partially kinase‐dependent

**Figure 3:**
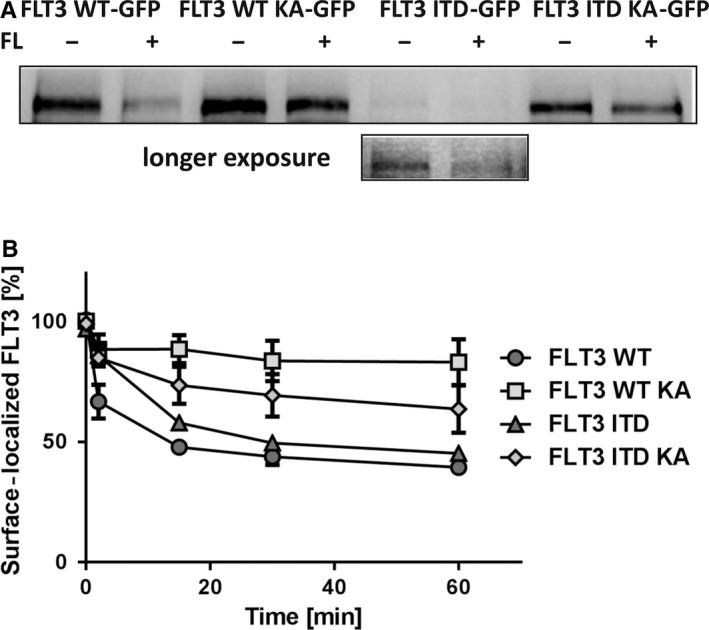
Ligand‐mediated internalization of FLT3 GFP proteins in 32D cells. A, FLT3‐GFP expressing 32D cells were incubated for 2 h with 10 nmol/L AC220 or left untreated and pre‐incubated with 20 μg/mL CHX prior to ligand stimulation. Cells were stimulated with 100 ng/mL FL 15 min at 37°C or left untreated. Cells were incubated with NHS‐biotin to label FLT3 located at the cell surface. After cell lysis, biotinylated FLT3 was precipitated with NeutrAvidin beads. Amount of biotinylated FLT3 was analysed by immunoblotting. Data show a representative experiment of two with consistent results. B, Kinetics of endocytosis of surface‐localized FLT3 GFP receptors in 32D cells expressing indicated FLT3 proteins. FLT3‐GFP expressing 32D cells were pre‐treated with 20 μg/mL CHX and stimulated with 100 ng/mL FL for the indicated time points. Surface‐exposed FLT3‐GFP receptors were stained with anti‐FLT3 ab89554 and anti‐mouse Cy3. Retained fluorescence was determined by flow cytometry. 100% value corresponds to FLT3‐GFP receptor population on the cell surface before FL stimulation. Error bars represent SD, n = 3

We first used surface biotinylation to assess FLT3 internalization of the different FLT3‐GFP hybrid proteins. As shown in Figure 3A, we detected similar features as with the endogenous receptors in human cell lines. FL stimulation led to efficient removal of WT FLT3 from the cell surface, which was attenuated but not completely blocked by kinase inactivation. FLT3 ITD was hard to detect at the cell surface, but the small amount visible upon prolonged blot exposure (lower panel) was reduced by ligand stimulation. Kinase inactivation in FLT3 ITD KA caused ready detectability at the cell surface, ligand stimulation reduced surface levels.

To more quantitatively study the kinetics of internalization, we used flow cytometry. An important issue was the choice of an antibody for these assays which would not compete with FL for binding. Among several tested antibodies, the here used antibody did not interfere with FL binding (data not shown). As shown in Figure 3B, FL stimulation of 32D cells expressing the different GFP fusion proteins led to internalization of all FLT3 variants. Active FLT3 WT‐GFP and active FLT3 ITD‐GFP were most efficiently internalized, FLT3 ITD KA‐GFP exhibited reduced but still robust internalization, while FLT3 WT KA‐GFP was measurably but only weakly internalized.

**Figure 4:**
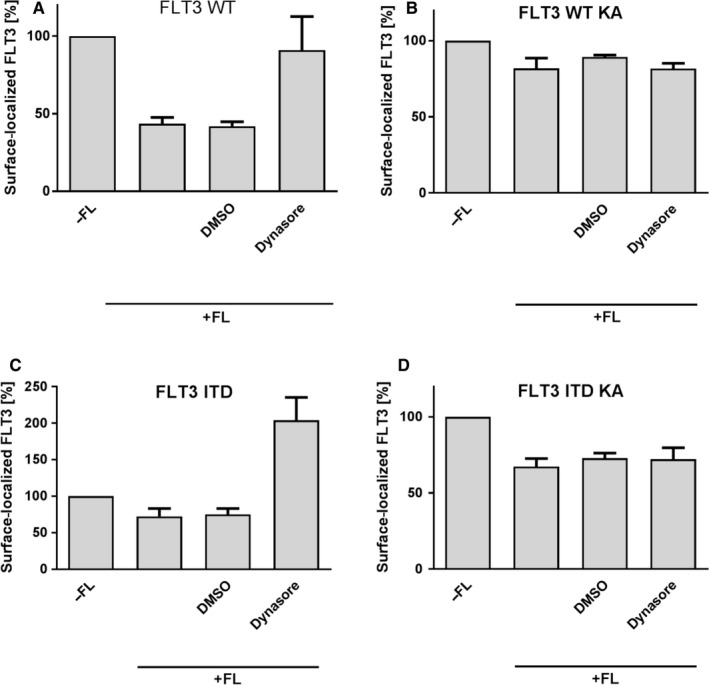
Detection of endocytosis route of FLT3‐GFP hybrid proteins. 32D cells expressing FLT3 WT (A), FLT3 WT KA (B), FLT3 ITD (C) or FLT3 ITD KA (D) GFP hybrid proteins were treated with 0.1% DMSO (solvent control) or 80 μmol/L Dynasore for 1 h at 37°C, as indicated. Receptor synthesis was blocked by pre‐incubating cells with 20 μg/mL CHX for 30 min at 37°C. After binding FL by incubation with 100 ng/mL FL for 2 h at 4°C, internalization was enabled by shifting cell temperature to 37°C for 15 min. Surface FLT3‐GFP receptor levels of cells were assessed by staining with anti‐FLT3 antibody ab89554 and subsequently with anti‐mouse Cy3 antibody, and were analysed by flow cytometry. Cell surface exposition of FLT3‐GFP hybrid proteins in absence of FL was set to 100%

We used this assay first to assess by which endocytotic pathway WT FLT3 is taken up in response to ligand stimulation. To this end, cells were left untreated or were treated with dynasore, which inhibits clathrin‐mediated endocytosis.^25^ In the presence of dynasore, internalization of FLT3 WT was almost completely inhibited (Figure 4A). Similarly, FLT3 ITD internalization was completely abrogated by dynasore (Figure 4C). Elevated surface localization of dynasore‐treated 32D FLT3 ITD‐GFP cells might be due to promoting accumulation of previously intracellularly retained FLT3 ITD molecules in the plasma membrane as consequence of known off‐target effects of dynasore.^26^ Thus, it can be concluded that kinase‐active receptor FLT3 WT or FLT3 ITD, respectively, are likely to get internalized by clathrin‐mediated endocytosis. Surprisingly, FLT3 WT KA or FLT3 ITD KA endocytosis was not abrogated in the presence of dynasore (Figure 4B+D). Thus, these data indicate that enzymatically inactive FLT3 KA receptor variants obviously use an alternative internalization route.

### 3.4 Degradation of FLT3

In order to address the question, if the enzymatic activity affects turnover of FLT3 proteins, degradation of the FLT3‐GFP hybrid proteins was monitored by quantification of the cellular GFP level. To prevent de novo‐protein biosynthesis, cells were pre‐incubated with cycloheximide (CHX). GFP‐tagged FLT3 proteins showed similar degradation kinetics than compared to the untagged counterparts as shown by immunoblotting or flow cytometric detection of total cellular FLT3 protein (data not shown). In the absence of its ligand, the FLT3 WT receptor showed a protein degradation rate which appeared somewhat higher than that of FLT3 ITD or of the enzymatically inactive FLT3 KA and FLT3 ITD KA variants (Figure S3A‐D). Immunoblotting of cellular FLT3‐GFP revealed similar degradation kinetics (Figure S4). Incubation of 32D FLT3 WT cells with FL promoted degradation, most efficiently during the first hour of FL treatment (Figure S3A and S4A). In order to assess if the slight constitutive phosphorylation of FLT3 WT (as shown in Figure S2B) stimulated degradation, kinetics of FL‐mediated protein decay was measured after addition of tyrosine kinase inhibitor AC220, however, similar FLT3 WT degradation was observed in the presence or absence of AC220 (Figure S5A). Decay of active FLT3 ITD was lower than that of FLT3 WT or of both FLT3 KA or FLT3 ITD KA. Degradation of FLT3 ITD was FL‐independent likely due to its predominant intracellular localization excluding a large fraction of FLT3 ITD from ligand binding. While degradation of catalytically inactive FLT3 WT KA was nearly not altered in response to cytokine treatment, half‐life of FLT3 ITD KA was shortened in response to incubation with FL (Figure 3B,D). AC220‐mediated abrogation of FL‐mediated activation of FLT3 WT caused a similar degradation kinetic as that of FLT3 ITD and FLT3 WT KA proteins (Figure S5B,C). Surprisingly, in the case of FLT3 ITD KA, a partial stabilization of the receptor could be observed upon AC220 treatment (Figure S5D), possibly through side effects of the inhibitor on other kinases.

## 4 DISCUSSION

Plasma membrane proteins, such as RTK, undergo constitutive turnover mediated by internalization and subsequent degradation.^27^ The canonical model of RTK endocytosis involves rapid internalization of RTK activated by ligand binding at the cell surface, and subsequent sorting of internalized ligand‐RTK complexes to lysosomes for degradation, or for recycling to the plasma membrane.^28^ In this study, we comparatively investigated the internalization of the RTK FLT3 and of its oncogenic counterpart FLT3 ITD using surface protein biotinylation of endogenous FLT3 and FLT3 ITD in human leukaemia cell lines, and laser scanning microscopy, flow cytometry, imaging flow cytometry and surface biotinylation using FLT3‐GFP hybrid genes stably expressed in murine myeloblast 32D cells. Analysis of FLT3 protein localization in the absence of the ligand FL confirmed knowledge about the activity‐dependent intracellular retention of constitutively active FLT3 ITD previously published.^8^, ^29^ As main finding of this study, we observed that FLT3 WT and surface‐bound FLT3 ITD exhibit very similar ligand‐induced internalization and degradation characteristics. Thus, our data support that aberrant FLT3 ITD localization and signalling relate mainly to an impaired biogenesis route and not to alterations in endocytosis. Internalization is more effective for active FLT3 variants and sensitive to dynasore suggesting that it occurs through the ‘classical’ clathrin‐mediated pathway. However, there is still remaining internalization under conditions of genetic or pharmacological kinase inactivation by yet unidentified mechanisms.

There were, however, a number of subtle differences in internalization and in particular in degradation kinetics of the different FLT3 variants analysed, which are of interest. Basal degradation of FLT3 WT was, compared to other FLT3 variants, most efficient. Ligand‐treatment stimulated it further. In the presence of the TKI AC220 FLT3 WT FL‐induced turnover was similar to degradation kinetics in absence of FL. Thus, it can be concluded that FLT3 phosphorylation promotes receptor degradation. This finding is in accordance with observations of Sargin et al,^6^ who found that stability of mature, complex glycosylated FLT3 ITD was lower than that of FLT3 WT. They in addition demonstrated that cytosolically retained immature high mannose for of FLT3 ITD was more stable than its WT counterpart. Increased half‐live of the immature FLT3 ITD form is probably correlated with the subcellular localization in ER‐near compartments. Alternative degradation pathways were previously identified for surface‐bound and intracellular FLT3 species.^30^ Recently, Todde et al found that FLT3 ITD adopts a more stable configuration that the native enzyme,^31^ which may also relate to lower degradation rates observed in the current study. Obviously, due to its internal localization, the half‐life of FLT3 ITD was independent of FL. In contrast, FL treatment moderately stimulated degradation of the kinase‐inactive FLT3 ITD KA. While FL cannot activate the kinase activity of this FLT3 ITD variant, it may still further promote its dimerization, despite that the ITD sequences already confer a constitutive dimerization capacity.^17^ Surprisingly, FL stimulation of FLT3 WT KA had a lesser effect on degradation suggesting that dimerization makes a lesser contribution in this case.

The canonical model of RTK endocytosis follows the understanding that ligand‐mediated phosphorylation of intracellular tyrosines is strongly linked to the initiation of endocytosis as shown for EGFR,^32^ or c‐KIT.^33^ In agreement with our findings, data for the EGFR^34^ and PDGFR‐ß,^35^, ^36^ another member of the RTK III family, indicated that internalization of the activated RTKs does not entirely depend on their phosphorylation. Kinase‐inactive mutant PDGFR‐ß also internalized, but with lower efficiency compared to the WT receptor.^36^ In this study, Pahara established receptor dimerization as essential and sufficient post‐ligand binding event in regulating PDGFR‐β internalization. Inhibition of PDGFR‐ß dimerization with ganglioside GM1 resulted in ligand‐mediated receptor phosphorylation but abrogated receptor internalization.^36^ Taken together, we currently hypothesize that ligand‐mediated FLT3 internalization is to some extent mediated by FL‐induced dimerization which may be further stimulated by a dimer‐promoting ITD in case of FLT3 ITD. Kinase activity and phosphorylation further promote degradation at least in case of FLT3 WT. Further investigation of the relative contributions of dimerization and kinase activity for internalization and degradation of FLT3 ITD may be of interest for a better understanding of the effects of kinase inhibitors in the absence and presence of FL.

In order to identify the endocytic pathway mediating the internalization of the FLT3 proteins, dynasore, a specific inhibitor blocking the GTPase dynamin which cuts off the clathrin‐coated pits from the cell membrane,^37^ was used to block clathrin‐mediated endocytosis. Dynasore abrogated the internalization of the enzymatically active FLT3 receptor variants FLT3 WT and FLT3 ITD in 32D cells indicating that both receptor variants were at least partially internalized via the clathrin‐mediated endocytotic route. This observation is in accordance with previous findings that clathrin‐mediated endocytosis is the major endocytic pathway of RTKs.^28^ Interestingly, internalization of catalytically inactive FLT3 WT KA and FLT3 ITD KA proteins was not affected by dynasore. It is possible that these receptor variants were not recruited in clathrin‐coated pits due to impaired ubiquitynation and therefore preventing this internalization route.^38^ It has been demonstrated that mutations of lysine residues in the kinase domain of the EGFR resulted in inefficient recruitment of EGFR into clathrin‐coated pits and inhibition of clathrin‐mediated endocytosis of the EGFR.^28^ Therefore, the effect of the macropinocytosis inhibitor LY294002 was investigated. Schmees and colleagues were able to show internalization of PDGFR via marcopinocytosis using LY294002.^39^ However, in the presence of LY294002, we did not observe abrogation of internalization of the enzymatically inactive FLT3‐GFP variants (data not shown).

Taken together, our data reveal that surface‐exposed FLT3 ITD follows similar endocytosis and degradation route compared to FLT3 WT. Thus, oncogenic activity of FLT3 ITD can be mainly linked to the retention of its biogenesis.

## CONFLICT OF INTEREST

The authors declare that they have no conflicts of interests. Content has not been published in similar or the same form elsewhere.

## AUTHORS’ CONTRIBUTION

All authors read and approved the final manuscript. JPM, SAB and FDB conceived and planned the experiments; FK, AK and JPM carried out FLT3 proliferation, signalling, localization, degradation and internalization experiments; KS and TT constructed FLT3‐GFP plasmids; KH and PR carried out and processed flow imaging experiments; HL carried out confocal microscopy.

## Supporting information

Supplementary Material
