# Supplementary material for: Wild‐type FLT3 and FLT3 ITD exhibit similar ligand‐induced internalization characteristics
Source: J Cell Mol Med. 2020 Mar 10;24(8):4668–76. doi: 10.1111/jcmm.15132 (PMC7176853; doi:10.1111/jcmm.15132)
Supplement: Supplementary file 1 — Supplementary Material [file JCMM-24-4668-s001.pdf]

Supplementary material

Material and Methods

Antibodies

Polyclonal rabbit anti-FLT3 antibodies C-20 (sc-479), anti-STAT5 (SC 417) were purchased from Santa Cruz Biotechnology (Heidelberg, Germany). Anti-P-Akt (Ser473) (193H12), anti-Akt (no. 9272), anti–P-p44/42 MAPK (Thr202/Tyr204, E10; no. 9106), anti-P-Stat5 (Y694, EPITOMICS no. 1208-1), anti-STAT5 (no. 9310) and anti-P-FLT3 (Y591) were from Cell Signaling Technology (Frankfurt, Germany). Anti-Erk1 (no. M12320) was purchased from BD Transduction Laboratories (Heidelberg, Germany). For flow cytometric detection of FLT3 CD135-PE was used. Due to interference with the FL binding site (data not shown) FL-treated cells were labelled with anti-FLT3 ab89554 and subsequently co-stained with anti-mouse Cy3 (Dianova, Hamburg, Germany).

Cloning of FLT3

Constructs for human FLT3 WT, FLT3 ITD and their respective K644A variants [1, 2] were fused in frame to eGFP and subsequently inserted in lentiviral vector piG2 [3] (kindly provided by Dr. C. Stocking). Hybrid FLT3-GFP genes were lentivirally packed and transduced into cytokine-dependent myeloid 32Dcl cells.

Cell culture and transductions

32Dcl3 cells were kept in RPMI 1640 medium containing 20 mM HEPES, supplemented with 10 % heat-inactivated FBS (BioWest, Essen, Germany), 1 mM sodium pyruvate, and 2.5 ng/mL IL-3 (PeproTech, London, United Kingdom). RS4-11 FLT3 WT and MV4-11 FLT3 ITD cells were kept in RPMI 1640 medium containing 20 mM HEPES, supplemented with 10 % heat-inactivated FBS and 1 mM sodium pyruvate. 32Dcl3 cells were stably transduced trice with the pseudotyped lentiviral particles expressing respective FLT3-GFP constructs in presence of 8 µg/ml polybrene (1,5-dimethyl-1,5- diazaundecamethylene polymethobromide, AL-118, # 10,768-9, Sigma-Aldrich, Deisenhofen, Germany) and subsequently sorted for similar GFP production using the BD AIRA sorting system (Becton Dickinson, Heidelberg, Germany).

Signaling analysis, immunoprecipitation immunoblotting

32D cells were starved from growth factors by incubation in serum- and cytokine-free RPMI 1640 with 1 mM sodium pyruvate, for 4 hours, followed by stimulation with 100 ng/mL FLT3 ligand (FL) at 37°C for 10 minutes before lysis. Lysis buffers were freshly supplemented with proteinase inhibitors and phosphatase inhibitors (2 mg/ml Leupeptin, 1% Aprotinin; 2 mg/ml Pepstatin A, 1mM PMSF, 1mM Pefabloc; 1mM sodium-orthovanadate; 1mM glycerol-phosphate), and cells were lysed on ice for 15 min. Aliquots of the cleared cell extracts were subjected to SDS-PAGE.

For FLT3 immunoprecipitation, lysis was done in 1 % NP40, 20 mM HEPES, pH 7.4, 0.15 M NaCl, 2 mM EDTA, and 1 mM EGTA. For detection of activated signaling proteins in cell lysates, RIPA buffer containing 1% NP40, 0.25% deoxycholate, 50 mM Tris pH 7.4, 0.15 M NaCl, and 1 mM EDTA was used. Lysis buffers were freshly supplemented with proteinase inhibitors and phosphatase inhibitors (25 mM NaF, 1 mM sodium orthovanadate, and PhosphSTOP; Roche, Mannheim, Germany), and lysis was allowed on ice for 15 minutes, before thorough vortexing and centrifugation. FLT3 immunoprecipitations were performed by incubating at 4°C overnight with anti-FLT3 antibodies, followed by incubation with Protein A- or Protein G-Sepharose beads.

Proteins were blotted to nitrocellulose or PVDF membrane (Millipore, Bedford, MA, USA) and probed with the indicated primary antibodies. After subsequent incubation with horseradish peroxidase conjugated secondary antibodies, the blots were developed using Western Lightning chemiluminescence detection (Perkin Elmer Life Sciences; Boston, MA, USA) and quantitatively evaluated using a CCD camera based system (LAS4000, Fuji, Düsseldorf, Germany). For quantification of specific phosphorylation, blots for phosphorylated proteins were stripped and subsequently reprobed with pan-specific antibodies. Specific phosphorylation was calculated as the ratio of the signals for phosphorylated protein to the signal for total protein detected.

Flow Cytometry

For quantification of surface-exposed FLT3, the corresponding 32D cells were washed with PBS and stained with PE-labeled anti-FLT3 (CD135) antibody (10 µl /10^6 cells) by incubating in 250 µl PBS at 4 °C for 30 minutes, followed by 2 washes with PBS containing 1 % BSA. For determination of total FLT3, 32D cells were washed once with PBS and subsequently fixed and permeabilized using ice-cold BD Cytofix/Cytoperm solution (BD, Heidelberg, Germany) for 20 minutes. Subsequently, the cells were resuspended in BD Perm/Wash buffer and stained with PE-labeled anti-FLT3 antibody diluted in the same buffer (10 µl /10^6 cells) for 30 minutes on ice, followed by 2 washes with BD Perm/Wash buffer.

Due to the interference of CD135-PE antibody and FL to bind FLT3 (our unpublished data), for FLT3 detection of ligand-treated cells were incubated FLT3 antibody ab89554: Thus, FL-treated 32D cells were washed with cold PBS and resuspended in 50 µl FLT3 mouse antibody (ab89554, abcam, 1:50) solution for 30 min on ice. Cells were subsequently washed twice with cold PBS and stained with 50 µl secondary antibody (anti-mouse Cy3, 1:50) for 30 min on ice. Cells were analyzed with a FACS Canto cytometer (Becton Dickinson, Heidelberg, Germany) using FlowJo software (BD Biosciences, Palo Alto, CA).

Imaging Flow Cytometry

All samples were processed using a 12 channel Amnis® brand ImageStreamX® Mark II (Luminex, Austin, Texas) imaging flow cytometer with excitation lasers 488 nm (200 mW) and 642 nm ((150 mW) at full power. 785 nm laser (70 mW) was used for sideward scatter analysis. Single color compensation controls for FLT3-GFP and WGA-Alexa Fluor 633 were acquired using the integrated software INSPIRE® (Luminex) for data collection. Image analysis was performed using image-based algorithms in the ImageStream Data Exploration and Analysis Software (IDEAS® 6.2.187, Luminex). Typical files contained imagery for 10,000 to 20,000 cells. The analysis was restricted to single cells in best focus. Single cells were identified by their intermediate size (area) and high Aspect Ratio (minor axis divided by the major axis) in comparison to debris (small area a range of aspect ratios depending on the shape of the debris) and doublets (large area and small aspect ratio). Out-of-focus events were excluded by using the feature Bright field gradient RMS via its image contrast. Living cells with an intact cell membrane (uniform texture of the WGA signal as measured by the H correlation mean feature) were selected to quantify the co-localization of FLT3 with the cell surface. The Bright Detail Similarity Score (BDS) R3 feature as way to quantify co-localization of two probes [4] was used to quantify accumulation of FLT3-GFP at the cell membrane. The BDS R3 feature is designed specifically to compare the bright image detail of two images and can be used.

BDS measures for co-localization of the fluorescently labeled cell surface marker (WGA-Alexa Fluor 633) and GFP-tagged FLT3 proteins were the log transformed Pearson's correlation coefficient of the localized bright spots with a radius of 3 pixels or less within the masked area in the two input images.

Correlated (same spatial location) or uncorrelated (different spatial location) bright spots in the images were set to a correlation coefficient 1 (correlation) and 0 (uncorrelated). The coefficient was log transformed to increase the range to between zero and infinity (0, inf). The BDS score was set to a threshold of 0.5.

Supplementary figures

Figure S1: Abrogation of FLT3 kinase activity by TKI AC220. FLT3 WT expressing RS4-11 and FLT3 ITD expressing MV4-11 cells were treated with 10 nM AC220 for 2 h during starvation. Subsequently, cells were stimulated for 2.5 min with 100 ng/ml FL. FLT3 was immunoprecipitated and the phosphorylation of Y591 or Y589 was analyzed with indicated site-specific P-FLT3 antibodies by immunoblotting.

Figure S2: Signaling activity of FLT3-GFP proteins A) Proliferation of 32D cells expressing FLT3 WT (●), FLT3 ITD (▲), FLT3 WT KA (■), FLT3 ITD KA (◆) or no FLT3 (▼)in absence or presence of the cytokines IL-3 or FL. Cells were cultured in RPMI 1640 medium containing 10 % FCS either without cytokines, with 100 ng/ml FL or 2 ng/ml IL-3 as indicated. Cells were counted every 24 h with counting beads using flow cytometry for 5 days. **B)** Signal transduction in 32D cells stably expressing the indicated human FLT3-GFP hybrid proteins. Cell were starved for 4 h in serum and cytokine-free RPMI 1640 medium and subsequently stimulated with 100 ng/ml FL for 5 min or left unstimulated. After stimulation, cells were lysed, separated by gel electrophoresis and analyzed by immunoblotting. Activation of FLT3, STAT5, AKT or ERK1/2 was analyzed using phospho-specific antibodies. Blots were re-probed with pan-specific antibodies. The blots shown are representative for at least three experiments with consistent results. **C)** Subcellular localization of FLT3-GFP proteins shown by immunofluorescence using confocal laser scanning microscopy. Counterstaining of the cells was performed with fluorescently labeled wheat-germ agglutinin to mark the cell surface, as indicated. Representative images are presented. (**D+E)** Graphs illustrate the ratio of surface-localized FLT3 receptors to total FLT3 receptor (**D**) or comparison of the PE signals of surface staining and GFP expression (**E**) of indicated FLT3 variants. **D)** Vital or fixed and permeabilized cells were labeled with PE-labelled CD135 antibody and processed by flow cytometry. As a negative control unstained cells were used. PE values of the surface population were compared to the PE values of the total receptor and normalized to FLT3 WT. Error bars represent standard deviation (SD). n = 3. **E)** Ratio of binding of PE-labelled CD135 antibody to cell specific GFP-level. PE values of the surface population were compared to the GFP values and normalized to FLT3 WT. Error bars represent SD. n= 3.

Figure S3: FL-dependent degradation of FLT3-GFP hybrid proteins. Cell specific GFP level of 32D cells expressing FLT3 WT (**A**), FLT3 WT KA (**B**), FLT3 ITD (**C**) or FLT3 ITD KA (**D**) GFP hybrid proteins in absence (open symbols) or presence (filled symbols) of FL. Cells were treated with 20 µg/ml CHX 30 min before stimulation to prevent de novo synthesis of FLT3-GFP. Cells were stimulated with 100 ng/ml FL or left untreated. At the indicated time points after FL addition samples were taken and GFP level was analyzed by flow cytometry. Error bars represent SD, n=3.

Figure S4: Activity and ligand-dependent degradation of FLT3-GFP hybrid proteins. 32D cells expressing indicated FLT3 variants were treated with 20 µg/ml CHX 30 min before stimulation with FL to prevent new synthesis of FLT3-GFP. Cells were stimulated with 100 ng/ml FL or left untreated as indicated. At indicated time points after FL addition samples were taken and level of FLT3 GFP proteins was analyzed by immunoblotting. The quantification of FLT3-GFP signals were normalized to ß-actin signals, respectively. A representative result of at least 4 times repeated experiments is presented.

Figure S5: Activity and FL-dependent degradation of FLT3-GFP hybrid proteins. Cell specific GFP level of 32 D cells expressing FLT3 WT (**A**), FLT3 WT KA (**B**), FLT3 ITD (**C**) or FLT3 ITD KA (**D**). GFP hybrid proteins in absence (filled circles) or presence (open circles) of FL or the TKI AC220 (20 µg/ml; filled triangles) to abrogate FLT3 kinase-activity prior stimulation. Cells were treated with 20 µg/ml CHX before stimulation to prevent new synthesis of FLT3-GFP. Cells were stimulated with 100 ng/ml FL or left untreated. At the indicated time points after FL addition samples were taken and GFP level was analyzed by flow cytometry. A representative experiment is 4 times repeated experiment is presented.

Supplementary references

1. Schmidt-Arras, D.E., et al., *Tyrosine phosphorylation regulates maturation of receptor tyrosine kinases.* Mol Cell Biol, 2005. **25**(9): p. 3690-703.
2. Mizuki, M., et al., *Suppression of myeloid transcription factors and induction of STAT response genes by AML-specific Flt3 mutations.* Blood, 2003. **101**(8): p. 3164-73.
3. Weber, K., et al., *RGB marking facilitates multicolor clonal cell tracking.* Nat Med, 2011. **17**(4): p. 504-9.
4. Beum, P.V., et al., *Quantitative analysis of protein co-localization on B cells opsonized with rituximab and complement using the ImageStream multispectral imaging flow cytometer.* J Immunol Methods, 2006. **317**(1-2): p. 90-9.

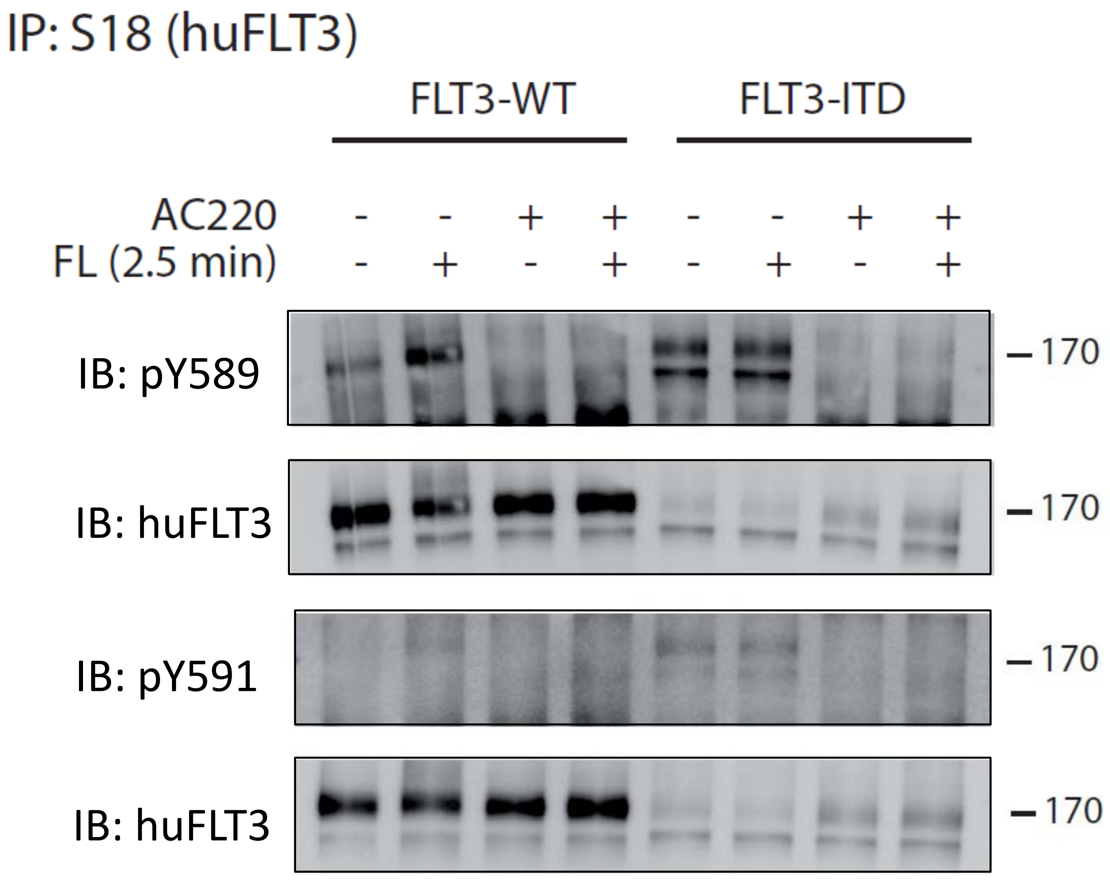
IP: S18 (huFLT3)
FLT3-WT
FLT3-ITD
AC220 - - + + - - + +
FL (2.5 min) - + - + - + - +
IB: pY589
IB: huFLT3
IB: pY591
IB: huFLT3
170
170
170
170

Figure S1

A

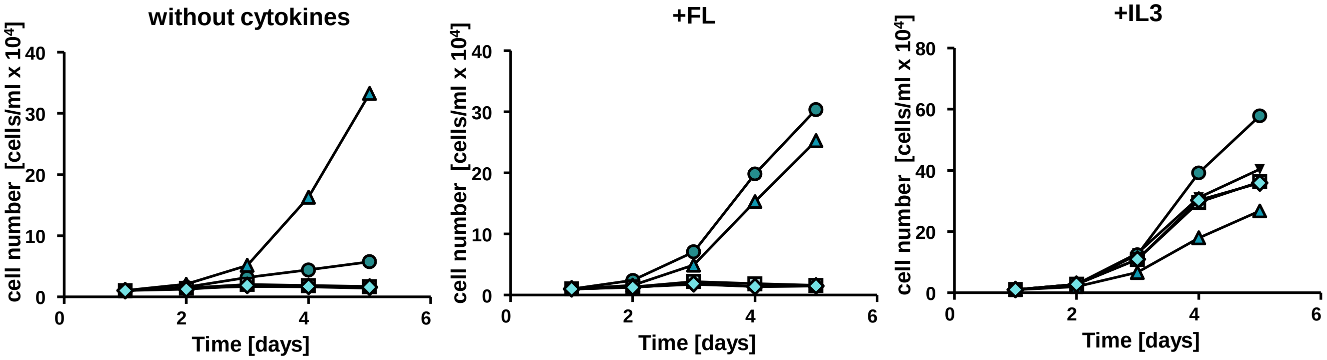

without cytokines
+FL
+IL3
cell number [cells/ml x 10⁴]
Time [days]
0
2
4
6
10
20
30
40
60
80

B

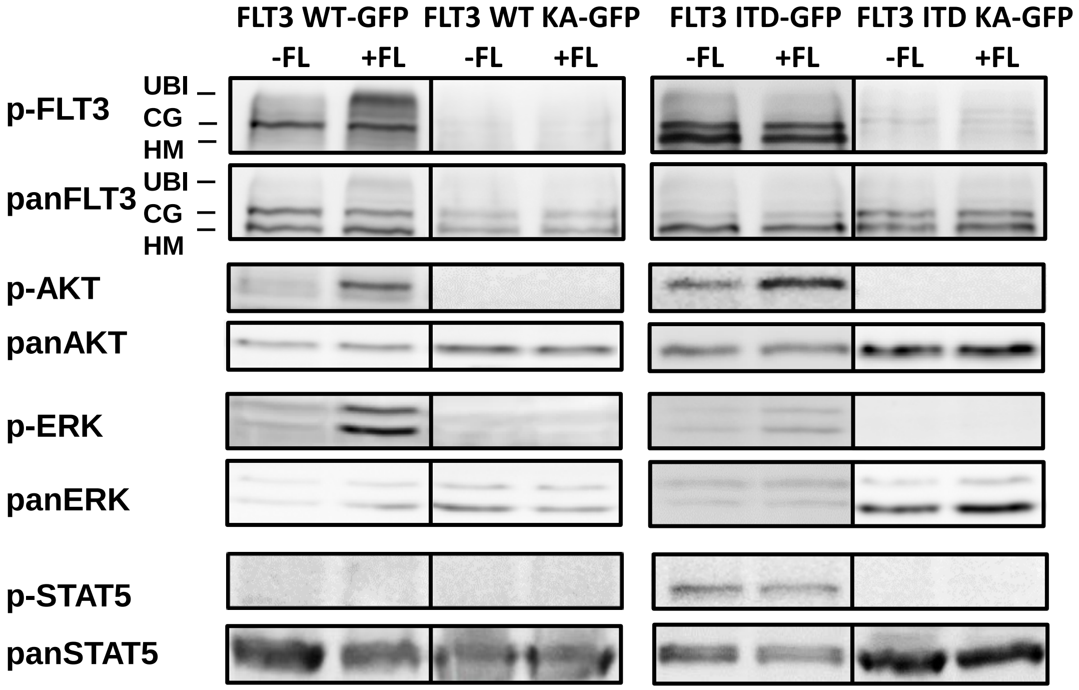

FLT3 WT-GFP
FLT3 WT KA-GFP
FLT3 ITD-GFP
FLT3 ITD KA-GFP
-FL
+FL
p-FLT3
panFLT3
UBI
CG
HM
p-AKT
panAKT
p-ERK
panERK
p-STAT5
panSTAT5

Figure S2

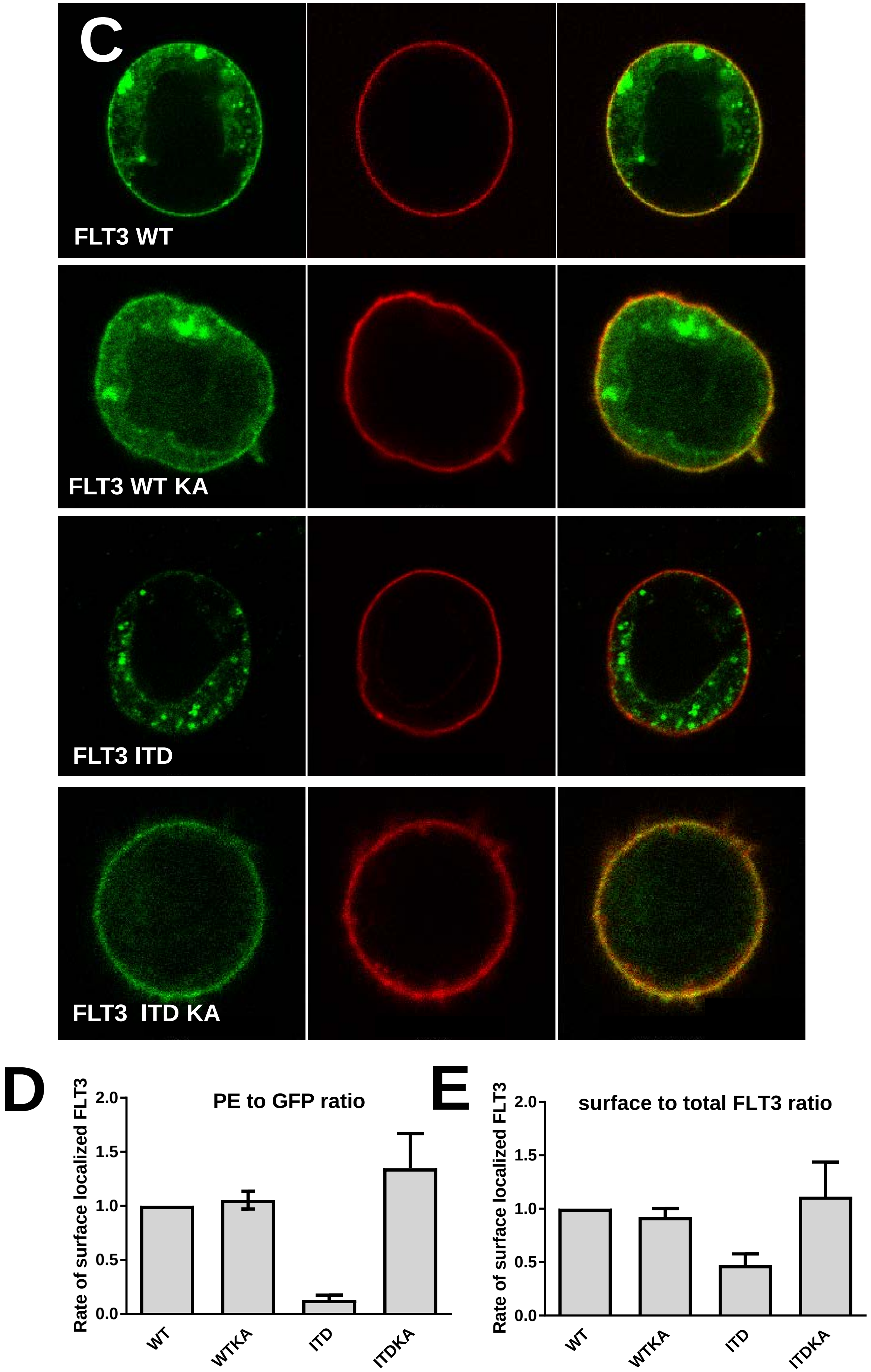
C
FLT3 WT
FLT3 WT KA
FLT3 ITD
FLT3 ITD KA
D
PE to GFP ratio
Rate of surface localized FLT3
2.0
1.5
1.0
0.5
0.0
WT
WTKA
ITD
ITDKA
E
surface to total FLT3 ratio
Rate of surface localized FLT3
2.0
1.5
1.0
0.5
0.0
WT
WTKA
ITD
ITDKA

Figure S2

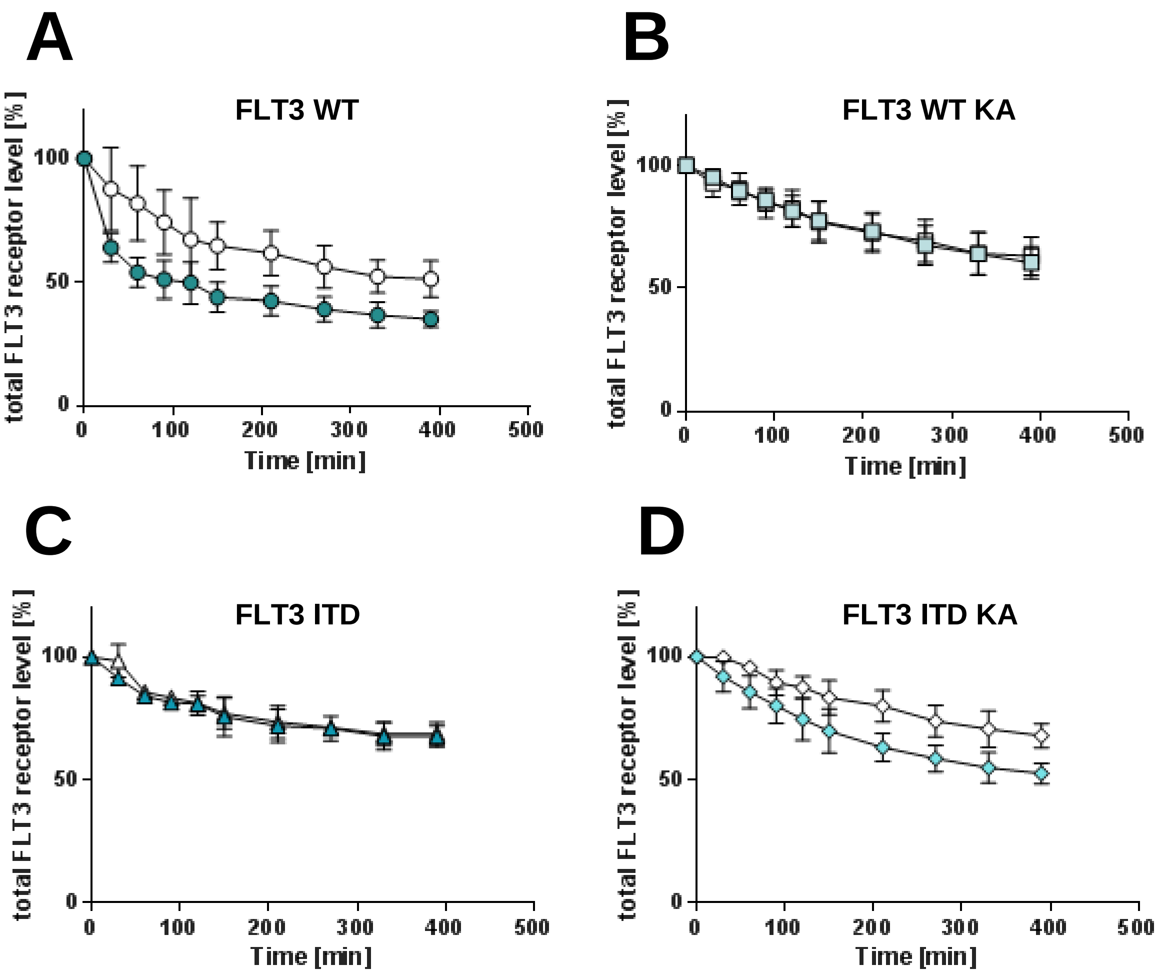
A
FLT3 WT
B
FLT3 WT KA
C
FLT3 ITD
D
FLT3 ITD KA
total FLT3 receptor level [%]
Time [min]
0
50
100
0
100
200
300
400
500

Figure S3

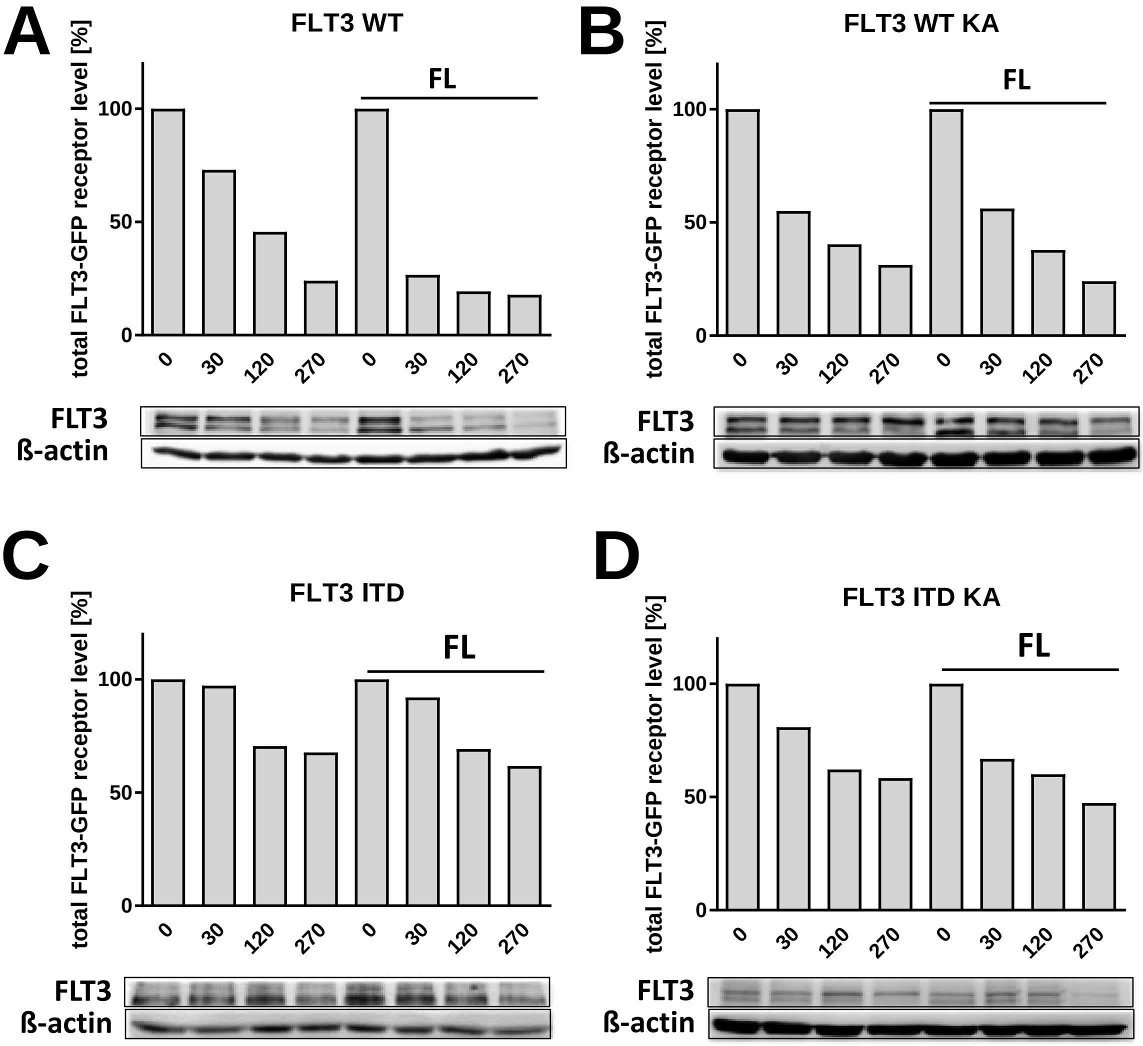

A
FLT3 WT
B
FLT3 WT KA
C
FLT3 ITD
D
FLT3 ITD KA
total FLT3-GFP receptor level [%]
FL
100
50
0
0
30
120
270
FLT3
ß-actin

Figure S4

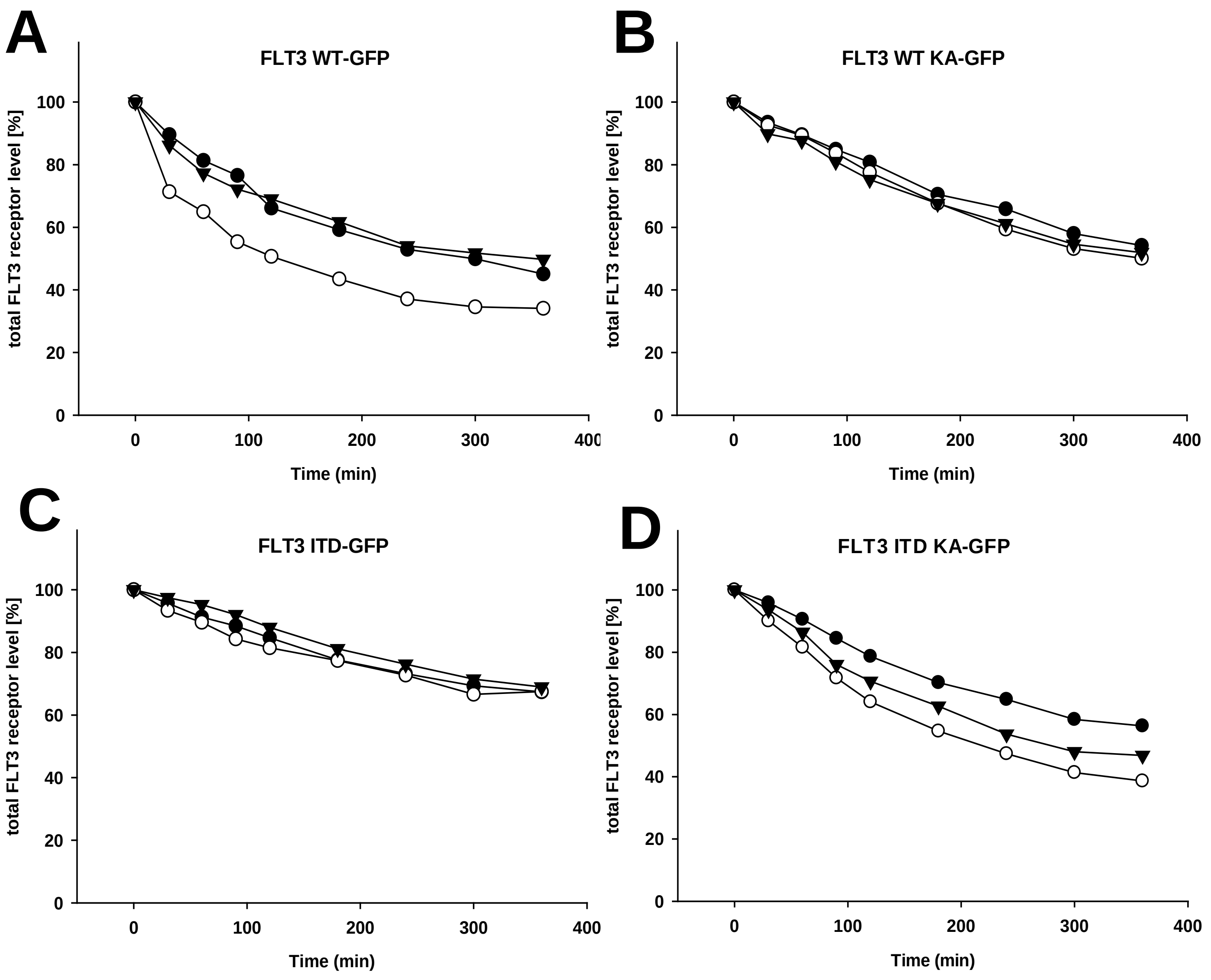
A
FLT3 WT-GFP
B
FLT3 WT KA-GFP
C
FLT3 ITD-GFP
D
FLT3 ITD KA-GFP
total FLT3 receptor level [%]
Time (min)
0
20
40
60
80
100
100
200
300
400

Figure S5
